# A neurological disorder presumably underlies painter Francis Bacon distorted world depiction

**DOI:** 10.3389/fnhum.2014.00581

**Published:** 2014-08-29

**Authors:** Avinoam B. Safran, Nicolae Sanda, José-Alain Sahel

**Affiliations:** ^1^Sorbonne Universités, UPMC Univ Paris 06, UMR_S 968, Institut de la Vision, INSERM, U968, CNRS, UMR_7210Paris, France; ^2^Neurosciences, Geneva University School of MedecineGeneva, Switzerland; ^3^Neurology Department, Hôpital FochParis, France

**Keywords:** Francis bacon, illusions, central metamorphopsia, dysmorphopsia, vision, art, neurological disorder

We read with interest the remarkable paper by Zeki and Ishizu (2013), on Francis Bacon's subverted representation of the body. On that occasion, we wish to share the results of an observation we recently made on Bacon's depicted deformities (Safran et al., 2012, ARVO poster,) that led us to consider Bacon's paintings to be the reflexion of a rare central perception disorder called dysmorphopsia (Kölmel, 1993) (see Figure 1). This conclusion was supported by Bacon's own detailed description of a perceptual phenomenon of dynamic distortion, progressively changing in magnitude and pattern, which he consistently experienced upon steady fixation.

**Figure 1:**
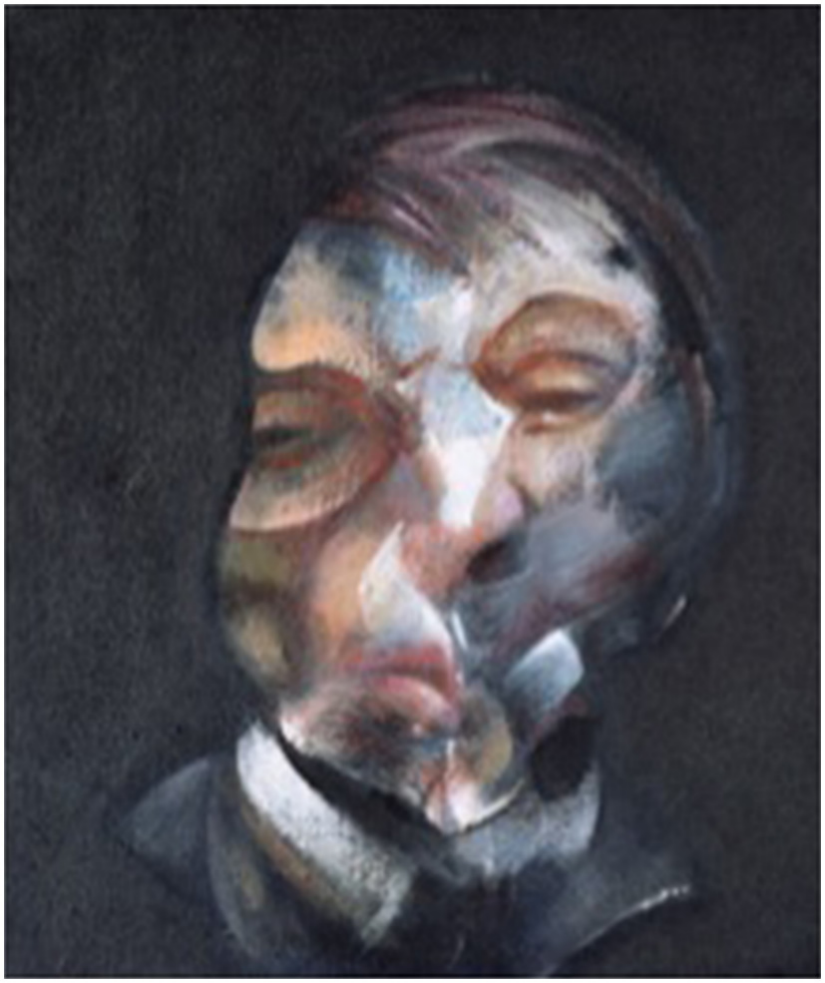
**Francis Bacon—Self-portrait 1971**. Centre Georges Pompidou, Paris, France. © The Estate of Francis Bacon/All rights reserved/ADAGP, Paris 2014.

Bacon's comments on his perceptual experience are found in published interviews (Russell, 1979; Sylvester, 1980; Clair et al., 1996; Peppiatt, 2008). In his discussions with renown art critic Jean Clair, Bacon reportedly stated: “When I am watching you talking—I don't know what it is - I see a kind of image, which constantly changes: the movement of your mouth, of the head, somehow; it keeps changing all the time. I attempted to trap this thing in the portraits.” (Clair et al., 1996, p 29). Another distinguished critic, David Sylvester, further quoted him as saying “[…] in my case, with this disruption all the time of the image—or distortion, or whatever you like to call it—it's an elliptical way of coming to the appearance of that particular body… And it needs a sort of magic to coagulate color and form so that it gets the equivalent of appearance, the appearance that you see at any moment, because so-called appearance is only retrieved for one moment as that appearance. (Sylvester, 1980, pp. 116–117). Still according to Sylvester, Bacon also acknowledged “I'm just trying to make images as accurately off my nervous system as I can. I don't even know what half of them mean” (Sylvester, 1980, p. 82).

Gross image distortion is a rare clinical manifestation of disordered higher visual function. It presents as episodes of dynamic, ever-changing deformities, a condition referred to as dysmorphopsia (Kölmel, 1993) or (central) metamorphopsia (ffytche and Howard, 1999). Usually, the image initially appears normal but undergoes illusionary transformation if looked at for any length of time. Visages appear distorted, contracted or expanded, often in a dynamic manner (Kölmel, 1993; ffytche and Howard, 1999); image may appear “cut up” and displaced (ffytche and Howard, 1999). It was associated with occipito-parietal (Trojano et al., 2009) and callosal (Cho et al., 2011) lesions. Dysmorphopsia might represent a variant of the “thin man phenomenon” (Safran et al., 1999), a perceptual distortion phenomenon occurring around focal field defects (Mavrakanas et al., 2009), as suggested by Ganssauge et al. (2012).

Striking similarities to Bacon's portraits are found in drawings produced by a patient with a parasagittal parieto-occipital meningioma, and right inferior homonymous defect (Mooney et al., 1965). This patient also experienced abnormal percepts featuring persons who demonstrated continuously changing distortions, similar to those described by Bacon. He stated the following: “[…] everything was always moving […]. The girl would start normal enough but rapidly her lips would get coarser, her mouth more open and her teeth long and pointed. […] men's faces going through similar contortions, very red and shiny under a fishlike eye, the lower part of the lid dragged down, showing a very bloodshot white” (Mooney et al., 1965).

Dysmorphic percepts apparently occurred over virtually the whole duration of his painting activities. With one exception, all 131 Bacon portraits assembled in the volume by Kundera (1996) were produced between 1961 and 1989, and showed consistent abnormalities in face depiction. Similar deformities were noted in other paintings, created from 1959 up to 1991 (Peppiatt, 2008). Previously (1949–1957), Bacon depicted distorted faces where salient abnormalities repeatedly consisted of frightening wide open mouth and large, pointed teeth (Russell, 1979; Sylvester, 1980; Kundera, 1996). Remarkably that specific deformity is one of the commonest features reported individual suffering from dysmorphopsia (Mooney et al., 1965; ffytche and Howard, 1999). Over the years, deformities in Bacon's portraits increased in forms and roughness.

The origin of Bacon's visual percepts is unknown. Painter's creativity has been ascribed to catalyzing effects of psychological disturbances generated by unhappy childhood (Peppiatt, 2008; Zeki and Ishizu, 2013). It is conceivable that cerebral injury had been caused during his childhood by violent blows reportedly inflicted by his father (Peppiatt, 2008). Moreover, Bacon suffered from asthma (Falliers, 1996). Cerebral hypoxic-ischemic lesions could have occurred during asthmatic attacks, which were reported to be “so severe that Bacon would lie in bed for days, blue in the face, struggling for each breath” (Peppiatt, 2008, p. 11). In addition, since Bacon has been prescribed morphine and stramonium to ease his bronchial spasms (Peppiatt, 2008), toxic factors (Vella-Brincat and Macleod, 2007; Glatstein et al., 2012) might be considered, although unlikely as not associated with pronounced systemic manifestations; in addition, distorted percepts upon sustained fixation consistently occurred over decades (Sylvester, 1980).

Influence on Bacon by fellow artists has been suggested (Sylvester, 1980, Peppiatt, 2008). Bacon was impressed by Picasso's fluidity of lines and inventiveness, which he discovered in 1927 at Rosenberg's Gallery. Bacon considered Picasso as the artist having come closer than anyone to “the core of what feeling is about”(Peppiatt, 2008, p. 46). Although most of works presented at Rosenberg's gallery were classical in style, and included no cubist compositions, it is conceivable that Bacon saw in some of the forms elaborated by Picasso a resemblance to his own perceptions, as also did other subjects affected by dysmorphopsia (Mooney et al., 1965; ffytche and Howard, 1999). He then felt that there was a way to transpose on canvas the reality—the very one reality that his senses presented him: “[…] I thought afterwards, well, perhaps I could draw as well” (Peppiatt, 2008, p. 46).

Bacon detailed description of distorted percepts point out the organic element in the grounds of his art. It might contribute to clarify Bacon's “enigma” (Peppiatt, 2008), and assist art analysts to revisit foundations of Bacon's major contribution to twentieth century painting. Furthermore, Bacon's observational and artistic talents provide us with invaluable insights into the perceptual phenomena of dysmorphopsia.

## Conflict of interest statement

The authors declare that the research was conducted in the absence of any commercial or financial relationships that could be construed as a potential conflict of interest.

## References

[B1] Cho J. Y. Moon S. Y. Hong K. S. Cho Y. J. Kim S. C. Hwang S. I. (2011). Teaching neuroImages: unilateral prosopometamorphopsia as a dominant hemisphere-specific disconnection sign. Neurology 76, e110 10.1212/WNL.0b013e31821d74a0 21624982

[B2] Clair J. Eschapasse M. Malchus P. (1996). Entretien avec Jean Clair, 1971, in Francis Bacon: Entretiens (Paris: Ed. Carré), 25–40

[B3] ffytche D. H. Howard R. J. (1999). The perceptual consequences of visual loss: ‘positive’ pathologies of vision. Brain 122, 1247–1260 10388791 10.1093/brain/122.7.1247

[B4] Falliers C. J. (1996). Asthma in the life of a modern british painter. Francis bacon (1909-1992). J. Asthma 33, 349–350

[B5] Ganssauge M. Papageorgiou E. Schiefer U. (2012). Facial dysmorphopsia: a notable variant of the “thin man” phenomenon? Graefes Arch. Clin. Exp. Ophthalmol. 250, 1491–1497 10.1007/s00417-012-1958-z 22389107

[B6] Glatstein M. M. Alabdulrazzaq F. Garcia-Bournissen F. Scolnik D. (2012). Use of physostigmine for hallucinogenic plant poisoning in a teenager: case report and review of the literature. Am. J. Ther. 19, 384–388 10.1097/MJT.0b013e3181f0cbb4 20861718

[B7] Kölmel H. W. (1993). Visual illusions and hallucinations. Baillieres Clin. Neurol. 2, 243–264 8137001

[B8] Kundera M. (1996). Francis Bacon: Portraits et Autoportraits. Paris: Les Belles Lettres

[B9] Mavrakanas N. A. Dang-Burgener N. P. Lorincz E. N. Landis T. Safran A. B. (2009). Perceptual distortion in homonymous paracentral scotomas. J. Neuroophthalmol. 29, 37–42 10.1097/WNO.0b013e318198ca37 19458575

[B10] Mooney A. J. Carey P. Ryan M. Bofin P. (1965). Parasagittal parieto-occipital meningioma. With visual hallucinations. Am. J. Ophthalmol. 59, 197–205 14268791

[B11] Peppiatt M. (2008). Francis Bacon Anatomy of an Enigma. London: Constable & Robinson Ltd

[B12] Russell J. (1979). Francis Bacon. 2nd Edn London; New York, NY: Thames & Hudson

[B13] Safran A. B. Achard O. Duret F. Landis T. (1999). The “thin man” phenomenon: a sign of cortical plasticity following inferior homonymous paracentral scotomas. Br. J. Ophthalmol. 83, 137–142 10396187 10.1136/bjo.83.2.137 PMC1722921

[B14] Safran A. B. Sanda N. Sahel J. A. (2012). Francis Bacon's distorted representation of faces presumably reflects occipital dysfunction. Invest. Ophthalmol. Vis. Sci. 53, E-Abstract 4846.

[B15] Sylvester D. (1980). Interviews with Francis Bacon. London: Thames & Hudson Ltd

[B16] Trojano L. Conson M. Salzano S. Manzo V. Grossi D. (2009). Unilateral left prosopometamorphopsia: a neuropsychological case study. Neuropsychology 47, 942–948 10.1016/j.neuropsychologia.2008.12.015 19136018

[B17] Vella-Brincat J. Macleod A. D. (2007). Adverse effects of opioids on the central nervous systems of palliative care patients. J. Pain Palliat. Care Pharmacother. 21, 15–25 10.1080/J354v21n01_05 17430825

[B18] Zeki S. Ishizu T. (2013). The “Visual Shock” of Francis Bacon: an essay in neuroesthetics. Front. Hum. Neurosci. 7:850 10.3389/fnhum.2013.00850 24339812 PMC3857539

